# Cytokines and cytokine receptors as targets of immune-mediated inflammatory diseases—RA as a role model

**DOI:** 10.1186/s41232-022-00221-x

**Published:** 2022-12-01

**Authors:** Tsutomu Takeuchi

**Affiliations:** 1 Saitama Medical University, 38 Morohongo, Moroyama, Iruma, Saitama, 350-0495 Japan; 2 Division of Rheumatology, Department of Internal Medicine, Keio University School of Medicine, Keio University, 35 Shinanomachi, Shinjuku, Tokyo, 160-8582 Japan

**Keywords:** Immune-mediated inflammatory diseases, Biological agents, JAK inhibitors, Cytokine, Cytokine receptor

## Abstract

Recent advances in our understanding in the immune-mediated inflammatory diseases (IMID) are explored and promoted by the targeted treatment. Among these targets, cytokines and cytokine receptors have become the good candidates for the drug development. In this review, the cytokine and cytokine receptors, which are approved in IMID, are overviewed, and modalities of the treatment, the role of cytokines and cytokine receptors in each disease, and the updated molecular information by modern technologies in rheumatoid arthritis as a role model are shown and discussed for the future perspectives.

## Introduction

Understanding the molecular and cellular processes involved in human disease is critical for exploring their underlying mechanisms. This understanding can also provide clues for the development of innovative strategies for diagnosis, monitoring, and treatment. Among human diseases such as cancer, infection, and metabolic disease, there exists a group of inflammatory diseases mediated by immunological mechanisms without any known etiology. These are designated immune-mediated inflammatory diseases (IMID) and include rheumatoid arthritis (RA), inflammatory bowel diseases (IBD), psoriasis (PS), and ankylosing spondylitis (AS) [1, 2]. Knowledge and understanding of the spectrum of IMID are growing, thanks not only to work in basic science and translational research but also to clinical trials and studies in clinical practice using biological and targeted drugs [2]. In particular, molecular targeted treatments have had an enormous impact on patients with IMID; they can even be considered game-changers against the burden of these diseases and in improving our understanding of them [2–4]. Among the myriad of possible molecular targets, numerous drugs have been developed against cytokines and cytokine receptors and introduced into clinical practice, demonstrating that they are druggable and reasonable targets. In this review, I summarize the growing body of evidence about cytokines and cytokine receptors in IMID and treatments targeted at them. I also propose a simple model of the cytokine network in patients with RA, a typical and common IMID [5].

## Structure of cytokine and cytokine receptor families implicated in IMID

This section will review the cytokines and cytokine receptors against which efficacious and safe drugs have been developed for IMID in clinical trials and clinical practice [6].

**Fig. 1:**
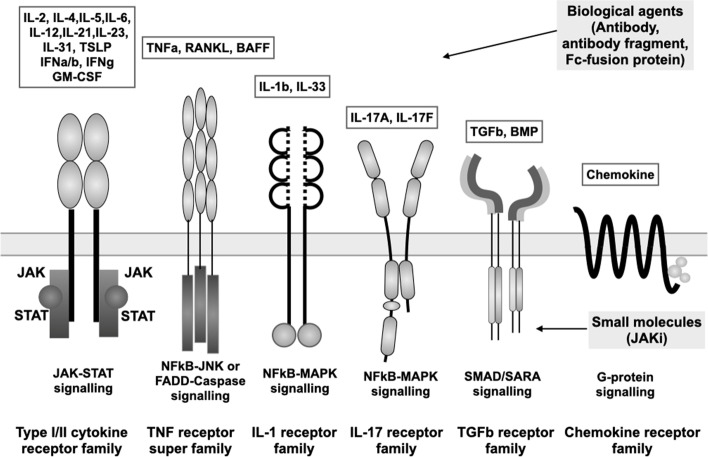
Cytokines and cytokine receptor families involved in immune-mediated diseases. White boxes in the top part of the figure list the cytokines implicated in IMID, which bind the cytokine receptors schematically depicted below. Horizontal gray bar represents the cell membrane. Under the cell membrane, signaling systems located downstream of the cytokine receptors are indicated. Gray boxes on the right list biological agents located outside the cells (top) and JAK inhibitors located inside the cells (bottom)

Figure 1 provides a schematic of the main cytokine receptor families, namely the cytokine receptor type 1 and type 2 family, TNF receptor superfamily, IL-1 receptor family, IL-17 receptor family, TGF-β family, and chemokine receptor, along with their ligands and their main mode of signal transduction. In the type 1 and type 2 cytokine family, the WSXWS motif, which plays a role in receptor folding through two tryptophan residues, is present in the type 1 cytokine receptor but is missing from the type 2 cytokine receptor for type 1 (IFNα/β), type 2 (IFNγ), type 3 (IFNλ) IFN, and IL-10 [7].

The TNF superfamily consists of more than 20 members, most of which have trimeric structures and use adaptor proteins such as TRAD/TRAF/TRIF/FADD for downstream signaling [8]. The TNF receptor plays a role in apoptosis and inflammation. Among the many superfamily members, TNF-α receptor and BAFF receptor and their ligands are two of the most important targets in IMID. Although both TNFR1 (TNFR1A, p55) and TNFR2 (TNFR1B, p75) can bind the TNF-α homotrimers as a ligand, only TNFR1 contains the death domain, which leads to apoptosis [9]. Meanwhile, the BAFF receptor (TNFR13 C) and TACI (TNFR13B) can bind BAFF homotrimers and transmit a signal to maintain mature B-cell survival.

IL-1R consists of IL-1R1 and IL-1R2, which are decoy receptors. IL-1R1 is cleaved or alternatively spliced to form soluble IL-1R, inhibiting the interaction between IL-1 and IL-1R. The IL-17 receptor family consists of 5 members (IL17RA, IL-17RB, IL-17RD, and IL-17E), and the functional receptor is a heterodimer consisting of IL-17RA and combinations of the other members [10]. TGFβR consist of TGFβR1 (ALK5), TGFβR2, and TGFβR3 (β-glucan), with TGFβR1 and TGFβR2 binding strongly to TGFβ1 but showing lower affinity to TGFβ2 [11].

Meanwhile, biological agents such as monoclonal antibodies, antibody fragments, and Fc-fusion proteins can access targets on the outside of cells. The introduction of these agents into clinical practice has revolutionized the treatment of disease, enabling more efficacious inhibition of disease progression [3, 4, 6]. These agents have made it possible to achieve higher-level goals such as remission, which is now a realistic goal of treatment [3, 12, 13]. More recently, orally available small molecules that inhibit the Janus-associated kinase (JAK)-signal transducer and activator of transcription (STAT) signaling pathway inside cells have been developed, and five JAK inhibitors (JAKi) have now been approved for the treatment of IMID [14].

## Appropriate cytokine and cytokine receptor targets in IMID

Table 1 summarizes the cytokine and cytokine receptor targets against which efficacious and safe drugs have been tested in clinical trials and marketed for IMID [15]. Each row lists the results for a cytokine and its receptor: TNFα and TNF receptor in the first row, followed by IL-1 and IL-receptor antagonists in subsequent rows, and so on. Each column shows the target disease, ranging from RA on the left to COVID-19 on the right. In addition to the disease, the possibility of detecting autoantibodies is also shown below the disease.

**Table 1:**
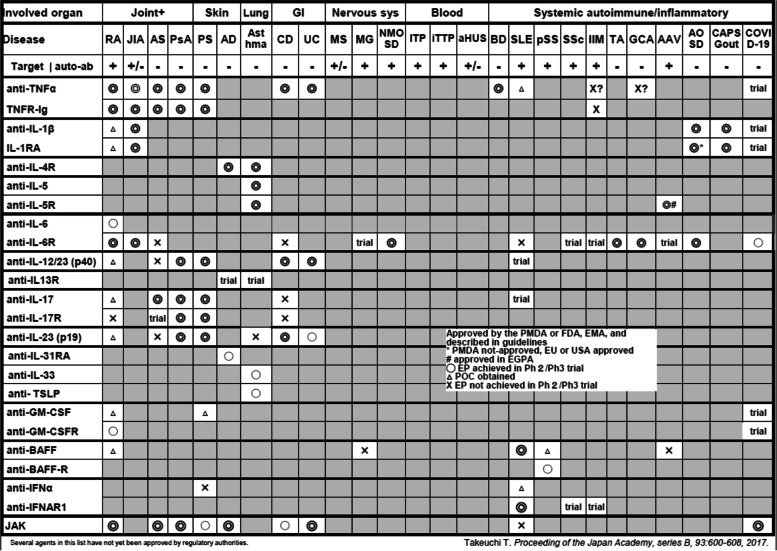
Cytokine/cytokine receptor targets in immune-mediated inflammatory diseases 2022

As shown in Table 1, drugs that target TNF and its receptor are approved and used in a wide variety of diseases including RA, juvenile idiopathic arthritis (JIA), AS, axial spondyloarthritis (SpA), PS, psoriatic arthritis (PsA), IBD, and Behcet’s disease (BD). The benefits of anti-IL-1 treatment were first revealed by strong evidence in arthritis animal models, which indicated its effectiveness in the prevention and amelioration of arthritis and joint destruction [16]. Additionally, clinical trials have proven that an IL-1 receptor antagonist (IL-1RA) is significantly more effective than placebo in patients with RA [17]. However, the clinical efficacy of these IL-targeted treatments and their ability to prevent joint destruction are not comparable to those shown by anti-TNF monoclonal antibodies. As a result, RA treatment guidelines and recommendations do not currently list anti-IL-1 treatment [18, 19]. This example offers the important lesson that targets found in animal models are not necessarily appropriate or valid in human disease but may be useful for identifying prophylactic and pre-arthritis treatment strategies [20].

The landscape of IL-6 receptor-targeted therapies in IMID differs somewhat from that of TNF and TNF receptors [21]. Anti-IL-6R is indicated in RA, but not AS, axial SpA, PS, PsA, or IBD [22]. Instead, it is approved for Takayasu arthritis (TA) [23], giant cell arthritis (GCA) [24], and adult-onset Still’s disease (AOSD) [25], raising an interesting hypothesis that the role of TNF and IL-6 may differ among IMID based on their distinct roles in the innate and adaptive immune systems or autoantibody production.

Anti-IL-17 treatment was also initially considered a good candidate for treating RA based on studies in an arthritis animal model [16]. Again, its efficacy in human RA was lower than the active comparator, abatacept, which is already approved for the treatment of RA [25]. Surprisingly, however, anti-IL-17 treatment showed efficacy against PS, PsA, and AS in a number of clinical trials [26], indicating a role for IL-17 in the inflammation of the enthesis (connective tissue between tendon and its ligament), rather than the synovium, which is characteristics of RA [27]. In addition to being produced by Th17, IL-17 is also produced by γ/δT cells, innate lymphoid cells (ILCs), and granulocytes, which are detected at sites of inflammation in psoriatic skin [26, 28]. IL-23 plays a role in the induction of Th17 cells and anti-IL-23 is approved for IMID, similar to anti-IL-17 treatment [26, 28]. IL-23 is a heterodimeric protein, composed of IL-12B (IL-12p40) and IL-23A (IL-23p19), produced from dendritic cells and macrophages, while IL-23 receptor is composed from IL-12 receptor beta1 and IL-23R. This molecule is utilized a part of IL12, suggesting anti-p40 treatment such as ustekinumab (anti-IL12/23 monoclonal antibody) may lead to inhibit function of not only IL23 but also IL-12. Interestingly, anti-IL-23 (IL-23p19) treatment does not show significant efficacy against AS, raising the hypothesis that IL-17 and IL-23 have distinct pathogenetic roles in PS/PsA and AS/axial SpA [28]. Evidence suggests that tissue-resident γ/δ T cells produce a large amount of IL-17 and express IL-23R. While neutrophils also produce IL-17, Th-17 in peripheral blood and entheseal tissues from AS patients do not [29]. Thus, it may be reasonable to anticipate the use of anti-IL17 treatment to inhibit the actions of IL-17 produced by a long list of inflammatory cells, whereas anti-IL-23p19 treatment may not inhibit IL-23-independent IL-17 production from γ/δT cells, neutrophils, and innate lymphoid cells (ILCs) in AS. In contrast, anti-IL-12/23 (p40/p19) and anti-IL23p19 are efficacious against Crohn’s disease, whereas anti-IL-17 worsens the disease, suggesting a role for IL-23 in local inflammation in gut epithelial cells and a protective role for IL-17 in Crohn’s disease.

Of note, anti-IL-4R/IL-5R/IL-13, cytokines that are characteristic of the Th2 response, have also been shown to be appropriate targets in patients with asthma [30]. Several other indications for diseases with similar Th2-related characteristics have also been approved, including eosinophilic granulomatosis with polyangiitis [31]. Furthermore, a long list of targeted biologics including antibodies against IL-4R, IL-5R, IL-13, IL-33, and TSLP has been tested against atopic dermatitis (AD) [32]. Given that these cytokine receptors likely transmit signals through JAK-STAT, JAKi are extending their indications from RA to other IMID, including AS, PsA, PS, AD, and more [14].

The information for safety aspects of the molecular target treatment is enormously increasing. The signals are influenced by the era, regions, health insurance systems, medical resources, patient backgrounds, disease activity, comorbidities, co-medications, and so on. The potential risks between anti-TNF and mycobacterial infections such as tuberculosis, anti-IL-17 and fungal infection, anti-IL6R and perforation of diverticulitis, JAK inhibitors, and herpes zoster are one example of the safety signals detected in models, clinical trials, and real world. The rare signals may be difficult to detect by clinical trials with a limited number and a limited period of exposures and can be found in real world data such as post-marketing surveillance (PMS). In this regard, Japanese PMS demonstrated the overall tolerability of targeted treatment in RA and some safety signals such as tuberculosis and pneumocystis pneumonia [3]. More recently, randomized controlled trials comparing the safety of tofacitinib and TNF inhibitors had been conducted as PMS. The patients with moderate to active RA patients with age 50 and over 50, having at least one cardiovascular (CV) risk factors, were enrolled and the risks for major adverse cardiac events (MACE), cancers, thromboembolic events, and mortality. The primary endpoint comparing the non-inferiority of tofacitinib 5 mg twice a day and 10 mg twice a day versus TNF inhibitors (etanercept or adalimumab), in terms of MACE and cancers, was not met, since the upper limit of 95% confidence interval is exceeding the pre-defined upper limit 1.8 [33]. The results may raise the hypothesis tofacitinib may have an increasing risk for MACE and cancers, and warning was released from regulatory agencies such as EMA and FDA. At same time, the warnings for other JAKi were added since the class effects are not ruled out. The other possibilities including the off-target effects by the particular chemical compounds should also be tested. The real-world data should be accumulated for the potential risks for JAK inhibitors other than tofacitinib.

Although the clinical sequence of the targeted treatment such as the different targets by the biological agents and JAK inhibitors can be one of the hot topics in clinical practice, this may be beyond the scope of this review. For the American College of Rheumatology (ACR) guidelines [19] and European League against Rheumatism (EULAR) recommendations [18] in RA, biological agents and JAK inhibitors were positioned in the similar phase of the treatment algorithm, whereas the Japan College of Rheumatology (JCR) guidelines show biologics may be preceded over JAK inhibitors because of the evidence of safety and socio-economic reasons [34]. The ordered selection of the targeted treatment may be different in each IMIDs and refer to each guideline, which are much more relevant to clinical practice.

## Structure of biological agents

**Fig. 2:**
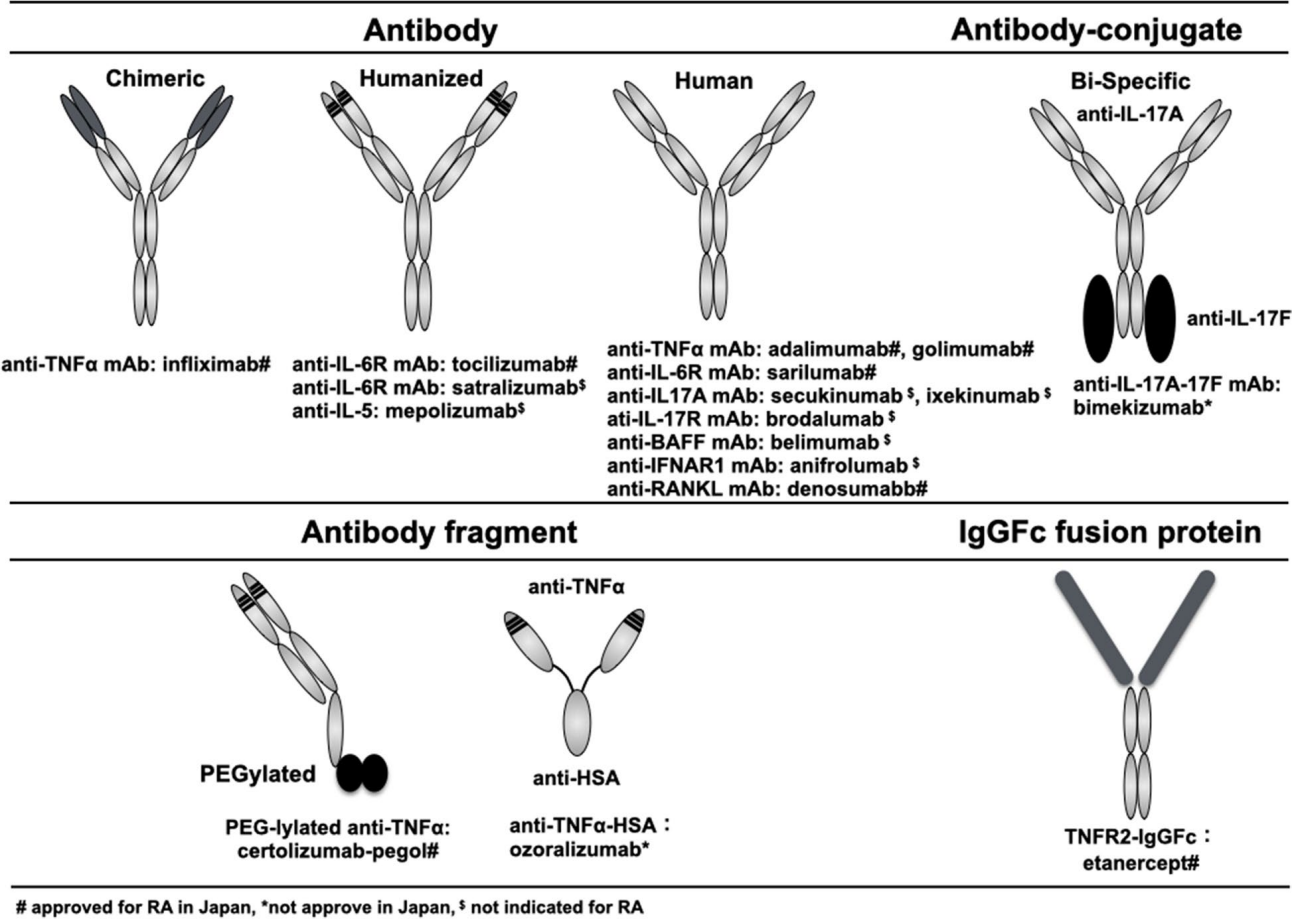
Structure of biological agents used to treat immune-mediated inflammatory diseases. Gray-colored regions in the chimeric and humanized antibodies indicate mouse proteins. #Approved in Japan for RA, *not approved in Japan, $not indicated for RA

The advent of hybridoma technology by Nobel laureates Drs. Kohler and Milstein in 1975 introduced monoclonal antibodies as a possible innovative treatment platform by which to target a single molecule with high specificity and affinity [35]. Initial clinical trials using monoclonal antibodies in RA patients, which used a mouse monoclonal antibody such as an anti-ICAM-1 antibody, showed that almost all recipients developed human anti-mouse antibodies [36], and that repeated treatment resulted in decreased efficacy [37]. Severe side effects were also reported, such as anaphylaxis [37]. Technical developments in molecular biology subsequently led to the development of “man-made” antibody molecules [38]. As shown in Fig. 2, chimeric monoclonal antibodies, humanized monoclonal antibodies, and fully human monoclonal antibodies have been developed using the phage display technique or by generating transgenic mice that express human immunoglobulins [39–42]. This ability to structurally change monoclonal antibodies from a chimeric to fully human form, along with appropriate administration with concomitant immunosuppressants such as methotrexate or one-shot glucocorticoids, has gradually reduced the immunogenicity of these antibodies [43].

In addition to full antibody molecules, antibody fragments and Fc-fusion proteins have also been generated with advantages such as more efficient production, much longer half-life and dual targets, among others (Fig. 2). Recently, anti-TNF antibody fragments linked to antihuman albumin were designed to prolong and stabilize blood concentrations of the antibody, with a phase II/III clinical trial demonstrating that the fragments were efficacious when administered every 4 weeks to active RA patients [44].

## Characteristics of JAKi

At present, five JAKi are indicated for IMID, including RA, AS, PS, PsA, ulcerative colitis, and AD [14]. All five molecules inhibit JAK activity by competitively blocking ATP binding sites on the JAK molecule, which are key to tyrosine kinase enzymatic activity [45, 46]. JAK consists of four different molecules, namely JAK1, JAK2, JAK3, and TYK2 [45, 46]. Tofacitinib and peficitinib have been reported to inhibit JAK1, JAK2, and JAK3 in an in vitro kinase assay, whereas baricitinib shows specificity for JAK1 and JAK2 [14, 46]. Upadacitinib inhibits JAK1, but JAK2 to a lesser extent, while filgotinib and its major metabolite appear to inhibit JAK1 [47]. Inhibition of distinct JAK molecules has only been demonstrated in primarily in vitro kinase and in vitro cytokine stimulation experiments. Thus, further research should be conducted in blood samples from patients treated with JAKi. However, it is obvious that these five drugs have different chemical formula and structure and exhibit distinct pharmacokinetic/pharmacodynamic profiles (Drug information and Kyoto Encyclopedia of Genes and Genomes (KEGG) (Table 2) [48]. While their chemical structures appear to be similar according to their mode of action as a competitor for ATP, as shown at the bottom of Table 2, their metabolism, route of excretion, metabolizing enzymes, and drug interactions differ, implying that these JAKi require different doses and dosing intervals (KEGG) (Table 2) [48]. Among the five JAKi approved for RA in Japan, peficitinib was developed in Japan [49]. Given the rationale that a higher dose leads to lower specificity, it is important to consider real-world data to determine efficacy and particularly safety.

**Table 2:**
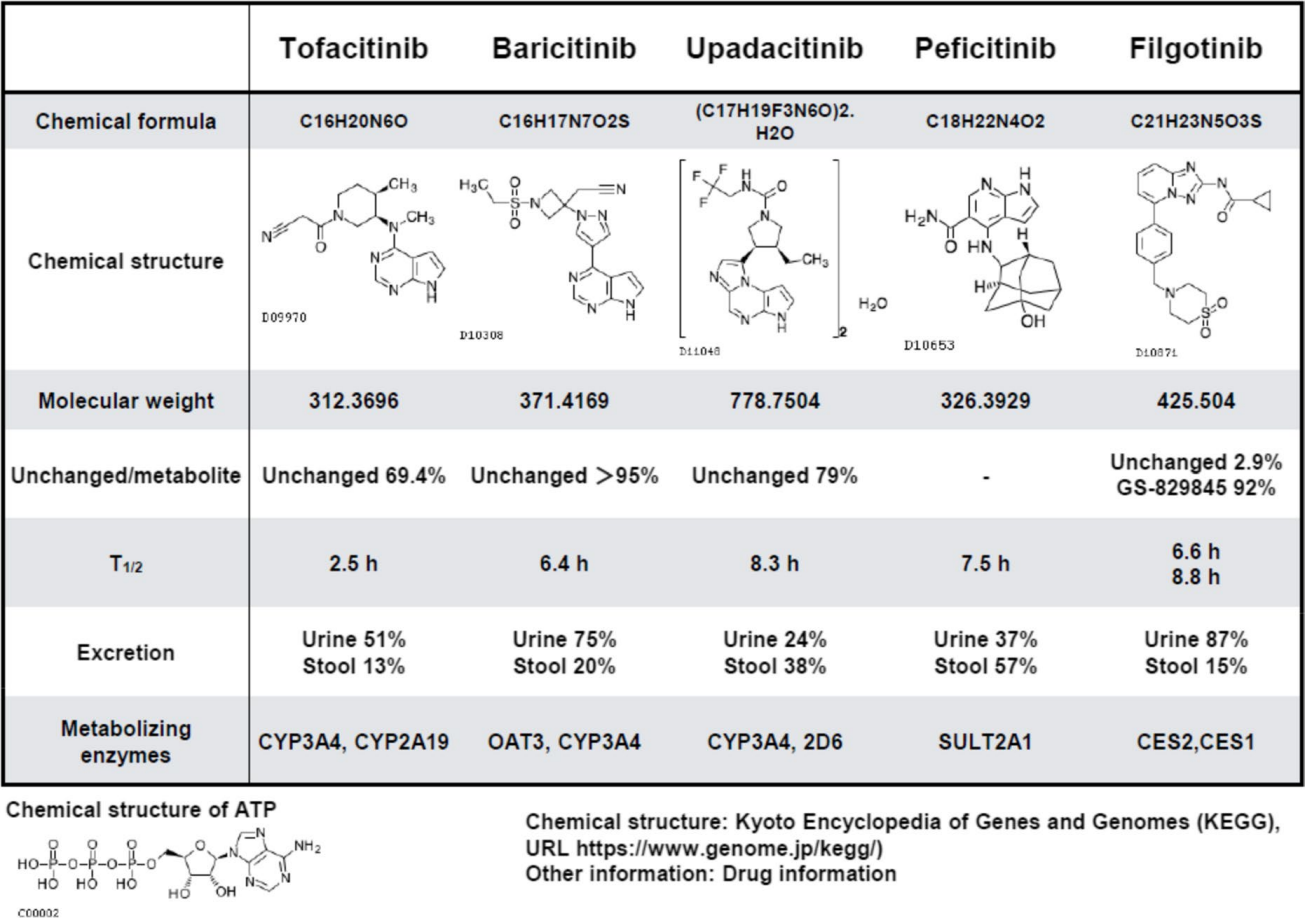
Structure of JAK inhibitors in immune-mediated inflammatory diseases

## Cytokine network in RA patients and targeted treatment

**Fig. 3:**
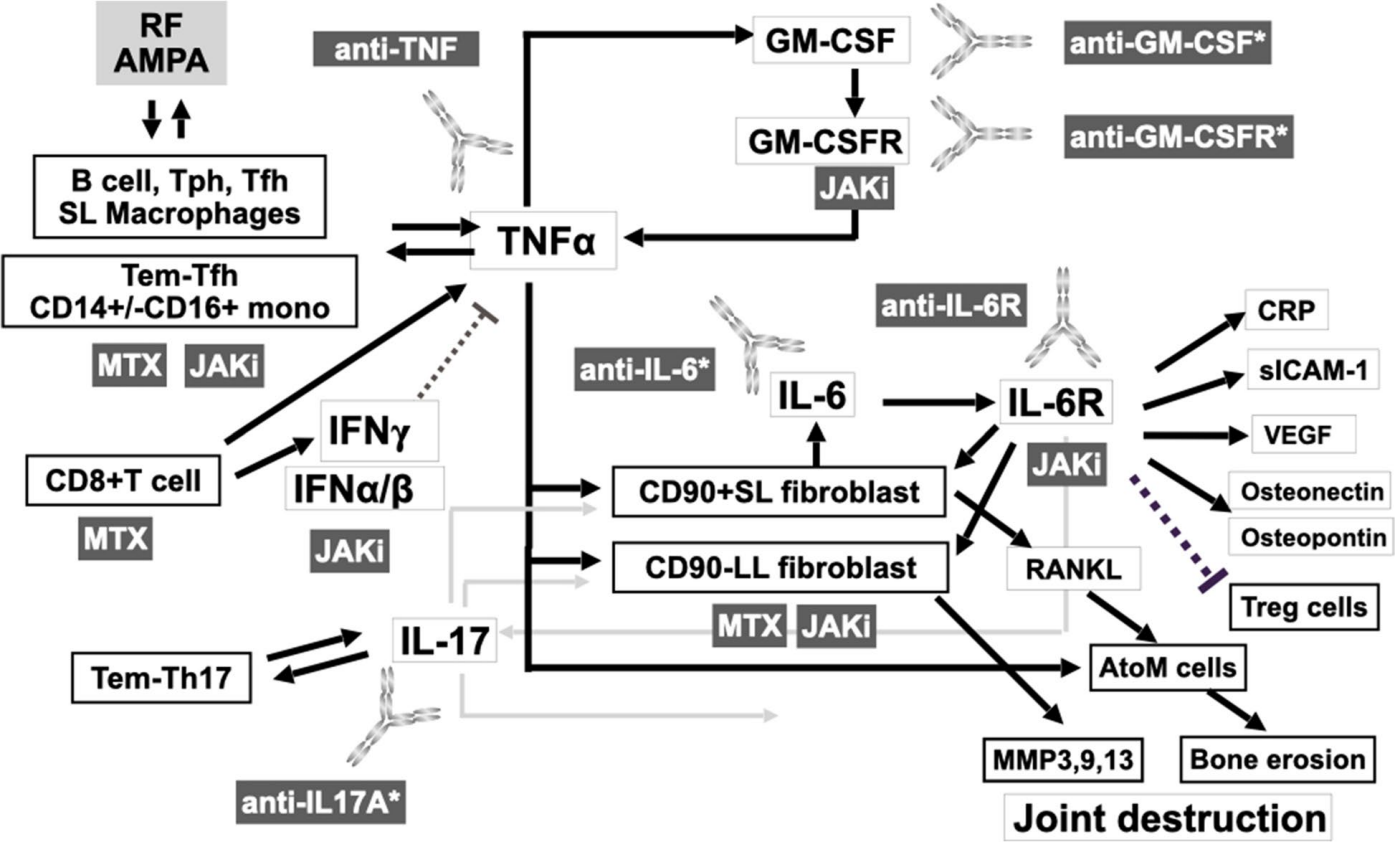
Working hypothesis of the hierarchical cytokine network in patients with RA. White boxes indicate cells. Light gray box indicates autoantibody. Dark gray boxes indicate targeted treatments and methotrexate (MTX). RF, rheumatoid factor; AMPA, anti-modified protein antibody; SL, sublining in synovial tissues; LL, lining in synovial tissues. *Not approved in Japan

This section will provide a comprehensive outlook on the network of cytokines and related receptors that are implicated in individual IMID [50]. These include the IL-6-TNF axis in RA and the IL-23-IL-17-TNF axis in PS, for example [51]. Here, one may wonder whether there are distinct groups of patients who exhibit a better response to anti-TNF or anti-IL-6R in RA, or to anti-IL-23, anti-IL-17, or anti-TNF in PS. Substantial efforts have been made to identify biomarkers to predict better response to targeted therapy [50]. For example, a previous study proposed that a myeloid biomarker, soluble ICAM-1, may be a predictor of response to anti-TNF, while the lymphoid biomarker CXCL-13 may be useful for predicting response to anti-IL-6R [52]. We have also identified unique transcriptomic patterns characteristic of responders and nonresponders to abatacept, infliximab, or tocilizumab among RA patients showing an inadequate response to methotrexate (MTX) [53]. However, cutoffs for these biomarkers are difficult to determine and validate as reliable and reproducible biomarkers may be required by a larger cohort with different patient background [50]. Furthermore, it is possible that several cytokines, rather than a single cytokine-cytokine receptor, may comparably contribute to the pathogenesis of inflammation, connected each other in individual patient [50, 51]. In this regard, a clinical trial examining different doses of infliximab in RA patients has already demonstrated that a hierarchical connection, but not multidirectional connection, exists between members of the TNF-IL-6 axis in RA [54]. This study found that only 2 h after infusion of the anti-TNF infliximab, serum IL-6 levels were significantly reduced, while those of IL-1β were not, indicating a hierarchical connection between TNFα and its upstream factors and IL-6 located downstream [54]. This cytokine network was also confirmed in the RISING clinical trial, in which RA patients with an inadequate response to MTX were randomized to receive placebo or infliximab 3 mg/kg, 6 mg/kg, or 10 mg/kg [55]. Plasma IL-6 levels significantly decreased after infliximab treatment, and the reduction was well correlated with treatment response [56]. Furthermore, it is interesting to note that levels of TNFα did not change for up to 1 year after tocilizumab-mediated blocking of IL-6R (personal communications). Rather than reducing levels of other cytokines, anti-IL-6R caused a rapid, substantial reduction in CRP and downregulation of osteonectin/osteopontin (personal communication). Surprisingly, we also observed an increase in the proportion of regulatory T cells (Treg). This increase in Treg by the anti-IL-6R tocilizumab was inversely correlated with the reduction in CDAI at 24 weeks and was significant at 52 weeks in RA patients [57], suggesting that Treg may be important for maintaining the control of disease activity. In this regard, a study proposed that, in mice, IL-6 directly suppresses the transcription factor FoxP3 [58], a master regulator of Treg. In addition, IL-6R transmits a signal to STAT3, which binds and competes with SATA5, activated through signal transmitted from CD122 (high affinity receptor for IL-2) to affect the transcription of IL-2, another important regulator of Treg [59]. These results provide a potential mechanism by which IL-6R inhibition increases Treg to normal levels in RA patients. This observation has also been reported for anti-TNF and MTX, but not for glucocorticoids or JAKi [60], which presumably block JAK3, interfering signal through common γ chain of IL-2 receptor signaling. These results provide a working hypothesis of the cytokine network of the TNF-IL-6 axis in RA (Fig. 3). Finally, double-positive and high titer status of autoantibodies such as rheumatoid factor and anti-CCP are associated with higher serum TNFα levels, suggesting the possibility that autoantibodies may interact with macrophages expressing Fc receptors and induce them to produce TNFα [61]. Theoretically, IL-6 can induce TNFα in a variety of human cells, proposing the IL-6-TNF axis as TNFα, a common pathway in IMID in RA [62]. The discrepancies may be due to the stage of the disease such as early or established, the species being tested such as human or animal model, and sites of focus such as systemic or joint, among others, warranting further investigation. Single-cell RNA sequencing analysis on synovial biopsy samples from untreated active RA patients could also provide important information to explain these discrepancies.

## Future perspectives for understanding the molecular mechanisms of RA and other IMID

There are several issues that warrant further consideration. First, among the mentioned molecular targets, it is important to consider the balance between the risks and benefits of their use in clinical practice. Some topics for discussion may include the prevention of disease [63], clinical sequence of the targeted therapy [61], monotherapy or combination therapy [64], tapering [65], and difficult-to-treat patients [66]. Among these, evaluating the balance between risks and benefits may be particularly important for determining the clinical sequence for guidelines or recommendations as discussed in the previous section. Although the molecular explanation for the observations and whether or not they are due to class effects or off-targets of the particular chemical compounds remain unclear, this clinical information may have a strong impact on treatment sequence.

Second, findings obtained from targeted treatments have primarily been examined in those with established disease. Thus, while the hypotheses proposed above, such as the hierarchical cytokine network in RA patients, may be applicable to established disease, they should be further investigated in those in the early stages of disease, such as MTX-naïve or even pre-disease stage RA patients [67–70].

Third, more research is needed to understand the molecular changes arising from targeted treatment in IMID. Muti-omics analysis of the peripheral blood of patients treated with MTX, anti-TNF, and anti-IL-6R has revealed the molecular signatures of RA patients, introducing new concepts of molecular remission and molecular residual signatures in RA [71].

Finally, use of emerging techniques such as single-cell spatial transcriptomics, mass cytometry, fate-mapping, and in vivo imaging to examine inflammatory tissues from both arthritis model animals and human clinical samples is providing an enormous amount of new information on IMID. These methods have enabled a more precise and clearer understanding of the molecular events occurring in synovial biopsy samples from untreated patients with active RA [72]. For example, we now know that TNFα is produced from synovial T cells, B cells, and macrophages, and that it transmits signals onto cells that express TNF receptor. Furthermore, the cells expressing TNF receptor have been identified as synovial fibroblasts and macrophages. On stimulation with TNFα, synovial fibroblasts produce IL-6 and matrix metalloproteinases and express RANKL, which leads to joint destruction, consistent with the efficacy of anti-RANKL treatment for inhibiting erosion in RA patients [73]. While these results suggest the presence of a hierarchical TNF-IL-6 axis in early and untreated active RA in the synovium, further information is needed to determine whether TNF or IL-6 may be first to hit this cytokine axis by obtaining information from biopsy samples after targeted treatment. In addition, recent research has discovered a new T-cell subset, designated T peripheral helper cells (Tph) [74], Thy-1(CD90)+/− subsets of fibroblasts [75], tissue-resident-protective macrophages [76], MerTk+/− monocytes subsets [77], and arthritis-associated osteoclastogenic macrophages (AtoM) [78] in synovial samples, and factors driving the process [79, 80], indicating that investigations focused on sites of inflammation can provide deep insight into disease pathogenesis. The molecular network models proposed by the information are depicted in Fig. 5.

**Fig. 4:**
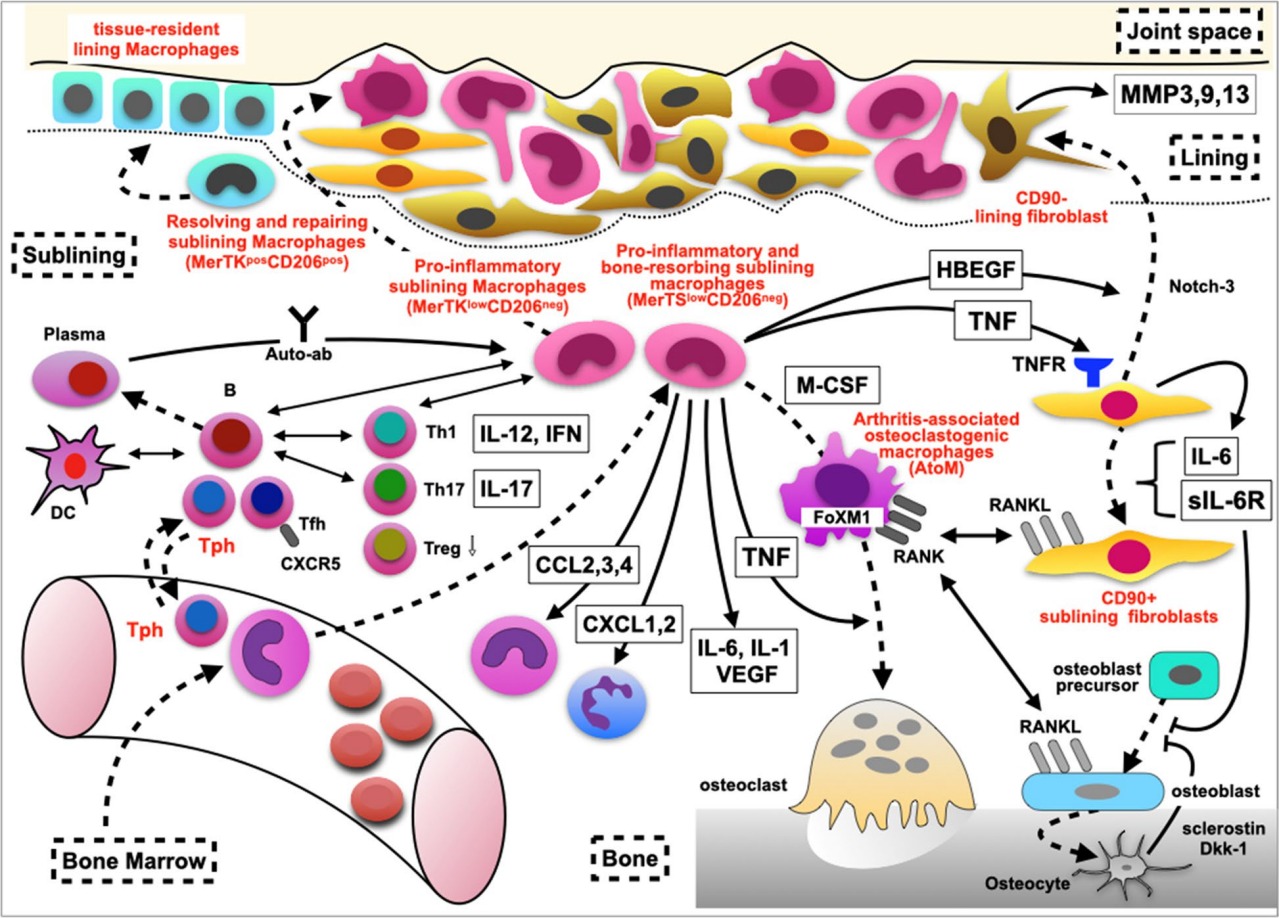
Hypothesized model of the molecular and cellular mechanisms underlying arthritis. Straw-colored region in the top part indicates joint fluid. Boxes enclosed by broken lines indicate the lining, sublining, bone, and bone marrow. Tissue-resident-protective macrophages [76] become activated and differentiate and move into the lining space. CX3CR1^low^ monocytes in the circulating blood produced in bone marrow migrate into the sublining space, where they become CXC3R1^high^ macrophages and produce cytokines and chemokines [77]. Some activated macrophages, stimulated by M-CSF, differentiate into precursors with FoxM1 and are designated arthritis-associated osteoclastogenic macrophages (AtoM) [78]. Boxes indicate cytokines and chemokines, and solid lines show their action on cellular targets. Broken lines show the shift or differentiation of a cell. Double-headed arrows indicate cellular interaction

Figure 4 summarizes the hypothesized molecular and cellular mechanisms underlying arthritis posed by recent reviews [81, 82]. Additionally, multi-omics analyses of peripheral blood with testing at multiple time points after targeted treatment may add useful supplemental information, such as enabling prediction of response to the targeted treatment and monitoring of disease activity. These advances in basic, translational, and clinical research will ultimately provide a more precise understanding of the pathogenesis of not only RA [63] but also other IMID [62].

**Fig. 5:**
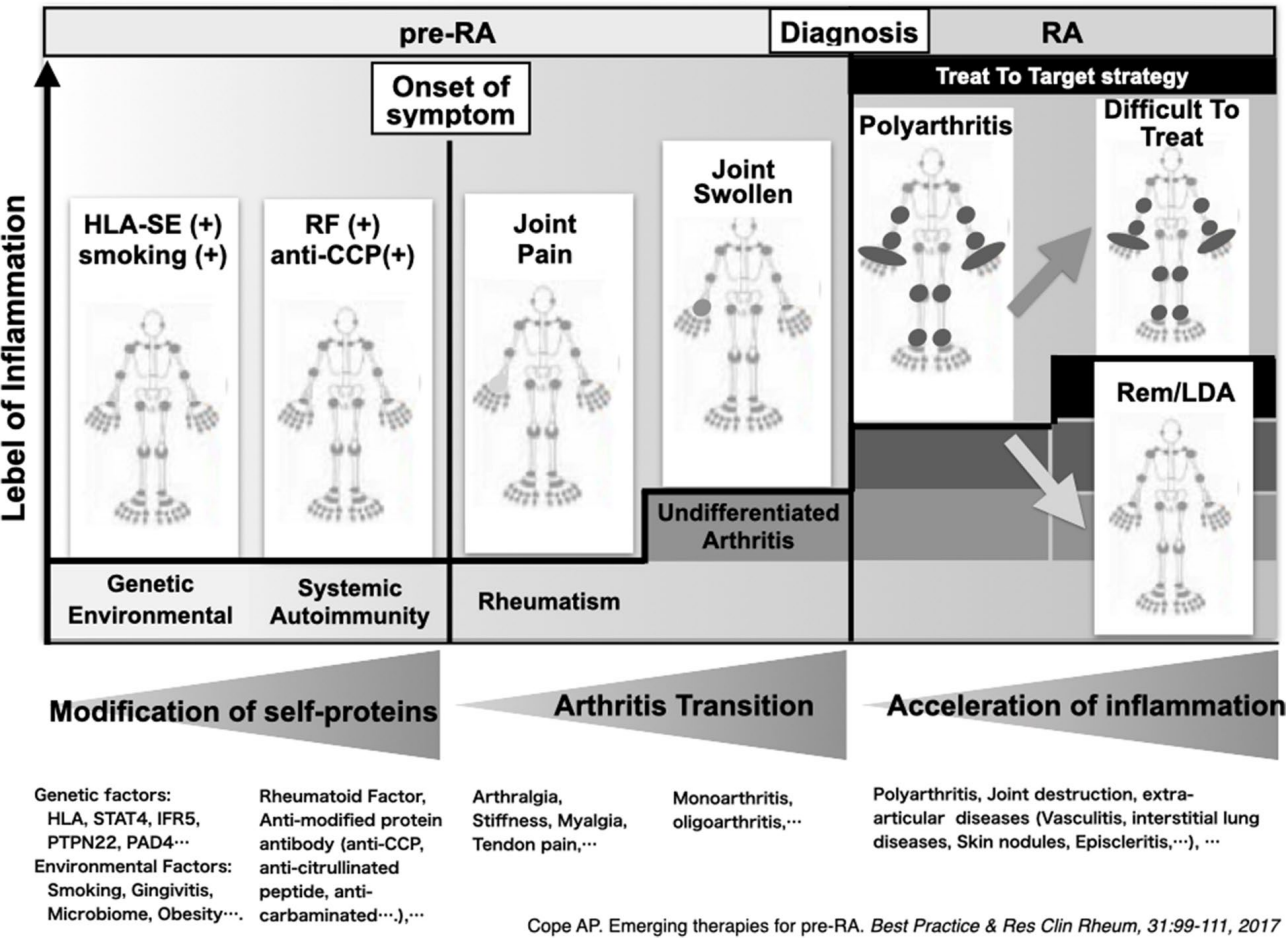
Pre-disease stage RA and RA [70]. The y-axis indicates the relative level of inflammation, and the x-axis indicates the relative time from the cause to the development of RA and the disease course after treatment

## Data Availability

Not applicable.
