# Correction: Cytokines and cytokine receptors as targets of immune-mediated inflammatory diseases—RA as a role model

**DOI:** 10.1186/s41232-022-00250-6

**Published:** 2022-12-28

**Authors:** Tsutomu Takeuchi

**Affiliations:** 1 Saitama Medical University, 38 Morohongo, Moroyama, Iruma, Saitama 350-0495 Japan; 2 Division of Rheumatology, Department of Internal Medicine, Keio University School of Medicine, Keio University, 35 Shinanomachi, Shinjuku, Tokyo 160-8582 Japan

**Correction: Inflamm Regen 42, 35 (2022)**

**https://doi.org/10.1186/s41232-022-00221-x**

Following publication of the original article [1], the authors would like to correct the reference information for the literature below:

48. Kyoto Encyclopedia of Genes and Genomes. https://www.genome.jp/kegg/. Accessed 27 Jul 2022.

The correct version is:

48. Kanehisa M, Goto S. KEGG: Kyoto Encyclopedia of Genes and Genomes. Nucleic Acids Res. 2000;28:27–30.

The original article [1] has been corrected.
